# From seropositivity to pathogenicity: causal evidence for autoantibodies in myasthenia gravis

**DOI:** 10.3389/fimmu.2026.2008202

**Published:** 2026-10-30

**Authors:** Fanxin Kong

**Affiliations:** 1 Department of Encephalopathy and Psychology, Shenzhen Traditional Chinese Medicine Hospital, Shenzhen, China; 2 The Fourth Clinical Medical College, Guangzhou University of Chinese Medicine, Shenzhen, China

**Keywords:** agrin, autoantibody, cortactin, LRP4, monoclonal antibody, myasthenia gravis, neuromuscular junction, pathogenicity

## Abstract

Autoantibody testing has expanded the serological spectrum of myasthenia gravis (MG), but detection of disease-associated immunoreactivity does not by itself establish direct pathogenicity. This Review examines how an MG-associated antibody moves from serological observation to mechanistic causality. We organize the evidence around three questions: whether the antibody signal is reproducible and biologically interpretable, whether circulating antibody can engage the native target and produce neuromuscular-junction dysfunction, and whether the resulting phenotype can be attributed to that antibody specificity. Acetylcholine receptor (AChR) and muscle-specific kinase (MuSK) antibodies provide mature causal benchmarks. Low-density lipoprotein receptor-related protein 4 (LRP4) illustrates established pathogenicity despite assay-dependent detection and an incompletely resolved balance between signaling interference, Fc-dependent effects, and local complement injury. Agrin has substantial biological and functional support, but independent sufficiency of human anti-agrin antibodies remains incompletely resolved. Cortactin provides a contrasting example in which repeated serological detection has not been linked convincingly to native target engagement or direct neuromuscular-junction dysfunction. ColQ, rapsyn, and collagen XIII remain unresolved for different reasons. Rather than assigning a numerical pathogenicity score, we identify the specific causal step that is missing for each antibody and match that uncertainty to the most informative experimental approach. This framework may improve interpretation of expanding antibody panels and help prioritize mechanistic studies that distinguish clinically useful biomarkers from directly pathogenic autoantibodies.

## 1 Introduction

Myasthenia gravis (MG) is one of the clearest examples of antibody-mediated neurological disease. Autoantibodies against the acetylcholine receptor (AChR), muscle-specific kinase (MuSK), and low-density lipoprotein receptor-related protein 4 (LRP4) impair neuromuscular transmission through distinct mechanisms. AChR antibodies can promote receptor loss, interfere with receptor function, and trigger complement-mediated postsynaptic injury; MuSK antibodies disrupt signaling required for postsynaptic organization; and LRP4 antibodies can interfere with the agrin-LRP4-MuSK pathway and engage Fc-dependent effector mechanisms (1–9). Their pathogenicity is supported by convergent molecular, physiological, and experimental evidence rather than by serological association alone.

The serological spectrum of MG extends beyond these established targets. Antibodies against agrin, cortactin, collagen Q (ColQ), rapsyn, collagen XIII, and other proteins have been described, particularly in patients without conventional AChR or MuSK antibodies (1, 10–18). Their evidentiary status is heterogeneous. Some recognize extracellular proteins directly involved in NMJ signaling, whereas others target intracellular or subsynaptic molecules that are less readily accessible to circulating immunoglobulin. Reported prevalence also varies across cohorts and assay platforms.

These differences create a practical interpretive problem. Repeated detection of an antibody does not necessarily mean that the antibody directly causes neuromuscular transmission failure. Conversely, a target may have substantial experimental support for pathogenicity even when routine serological detection is technically inconsistent. LRP4 illustrates the latter distinction: experimental and human tissue data support pathogenic involvement, whereas a 2026 multicentre comparison of cell-based assays found low prevalence and limited cross-assay concordance (8, 9). Diagnostic robustness and biological pathogenicity should therefore be assessed separately.

Agrin raises a different problem. Several cohorts have identified anti-agrin antibodies, including patients without AChR, MuSK, or LRP4 antibodies, yet other studies have failed to reproduce the signal (10–13). Active immunization against neural agrin can produce myasthenic abnormalities, and immunoglobulin (Ig; IgG, IgA and IgM) from a patient with both LRP4 and agrin antibodies produced weakness, electrophysiological impairment, and NMJ disruption after passive transfer (19, 20). Those experiments support pathogenic potential but do not fully isolate the effect of anti-agrin antibodies from coexisting specificities.

Cortactin illustrates an earlier causal gap. It is biologically relevant to actin remodeling and AChR clustering, and antibodies have been reported in subsets of seronegative MG. However, frequency and disease specificity have not been consistent across cohorts, and direct access of circulating antibody to native cortactin at the intact NMJ has not been established (13–15, 21).

Recent MG reviews have clarified the clinical utility of autoantibody testing and the biology of individual serological subtypes. The present Review addresses a different question: what evidence is required before an MG-associated autoantibody can reasonably be regarded as directly pathogenic? We organize that question around signal reliability, native target engagement and neuromuscular-junction function, and antibody-specific causal attribution (**Figure 1**). AChR and MuSK provide mature causal reference points; LRP4 represents established pathogenicity with unresolved assay and mechanistic questions; agrin represents incomplete causal attribution; and cortactin represents an accessibility and pathogenicity problem. ColQ, rapsyn, and collagen XIII are used as boundary cases in which different parts of the causal chain remain missing.

**Figure 1:**
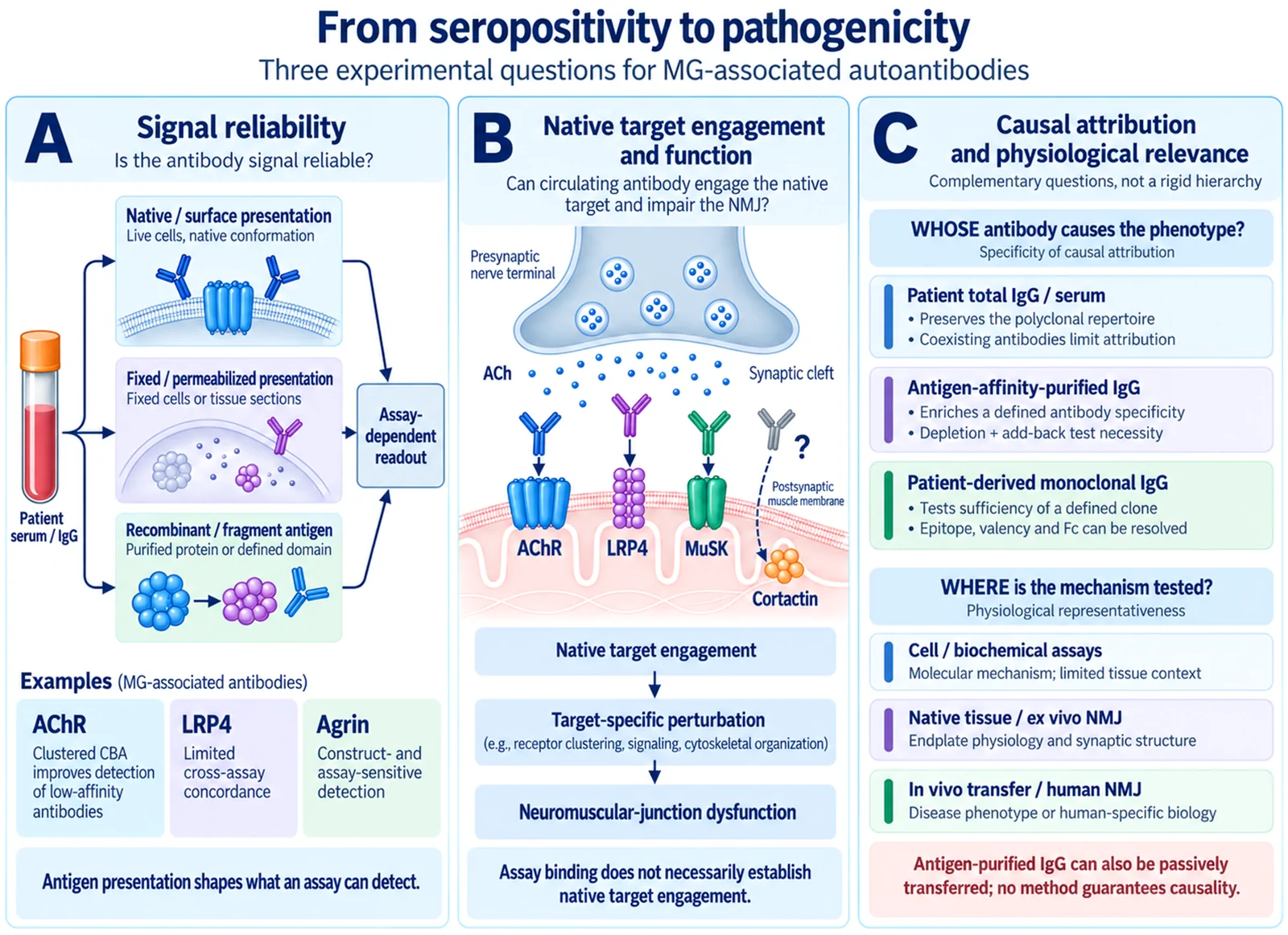
From seropositivity to pathogenicity: three complementary experimental questions for MG-associated autoantibodies. **(A)** illustrates antigen-presentation and positivity-threshold effects on serological readouts, whereas **(B)** distinguishes native target engagement, molecular perturbation, and neuromuscular transmission failure. **(C)** separates specificity of causal attribution (total patient immunoglobulin, antigen-affinity-purified immunoglobulin, and defined monoclonal antibodies) from the physiological context of the experimental system (cell-based assays, ex vivo tissue preparations, and *in vivo* transfer or human NMJ models). These dimensions are complementary rather than a rigid hierarchy: antigen-purified antibodies can also undergo passive transfer, and antigen-specific depletion followed by reconstitution provides additional evidence for causality.

### 1.1 Search strategy and scope

We performed a structured, targeted search of PubMed through September 2026 using combinations of “myasthenia gravis”, “autoantibody”, “AChR”, “MuSK”, “LRP4”, “agrin”, “cortactin”, “rapsyn”, “ColQ”, “collagen XIII”, “pathogenicity”, “passive transfer”, “monoclonal antibody”, and “neuromuscular junction”. Reference lists of key primary studies and recent reviews were screened for additional reports. Original studies addressing serological replication, antigen localization, antibody-specific molecular effects, neuromuscular transmission, passive transfer, or mechanism-directed intervention were prioritized, and contradictory or negative studies were sought where available. This article is a focused critical narrative review rather than a systematic review; no formal risk-of-bias assessment or meta-analysis was undertaken.

## 2 From serological detection to biological plausibility

### 2.1 Reproducibility across cohorts

For a newly reported specificity to carry mechanistic weight, its association with disease should be reproducible. This is particularly important for low-frequency reactivities in seronegative MG. With only a few borderline-positive samples, a modest change in the assay positivity threshold (for example, a different control-derived cutoff or interpretation of weak immunoreactivity) can move cases from positive to negative, changing the numerator and hence the reported prevalence disproportionately. Background reactivity, limited sample size, and cohort selection can further magnify such differences. These are methodological explanations, not a quantitative reanalysis of published assay data. Reproducibility alone does not establish pathogenicity, but an unstable serological signal weakens the basis for increasingly specific causal claims.

Cortactin illustrates the problem. An initial study reported cortactin antibodies in 19.7% of seronegative MG, with lower frequencies in seropositive MG, other autoimmune diseases, and healthy controls; 5.2% of healthy controls were nevertheless positive (14). A later study again reported enrichment in double-seronegative MG and suggested an ocular or relatively mild phenotype (15). In contrast, an independent 2021 study found cortactin antibodies in only three of 135 MG cases and in two controls; antibodies were not detectable in follow-up sera from the positive patients (13). The available studies therefore support recurrent cortactin-directed reactivity, but not a uniform or highly disease-specific serological signal.

Agrin shows a related but distinct pattern. A 2014 study identified anti-agrin antibodies in 7 of 93 MG sera and none of 80 healthy or neurological controls (10). A large Chinese study using a full-length agrin construct detected antibodies in 18 of 1,948 patients, including nine patients who were agrin-only positive (12). By contrast, the 2021 independent cohort of 135 MG patients detected no agrin antibodies (13). These prevalence estimates cannot be assumed to measure an interchangeable analyte: studies differed in antigen constructs and epitope presentation, sample processing, assay platform, positivity criteria, and composition of the tested cohorts. In particular, the full-length agrin cell-based assay used fixed and permeabilized transfected cells (12), whereas other approaches used different antigen presentations. These differences can change whether conformational, truncated, or intracellularly exposed epitopes are detected; they preclude direct cross-study equivalence without head-to-head testing of the same specimens.

### 2.2 Assay architecture changes the meaning of a positive result

Antibody assays do more than measure seroprevalence. They determine which epitopes are displayed, whether conformational structure is retained, and whether normally inaccessible antigens become exposed. Clustered AChR cell-based assays provide a useful benchmark. By expressing AChR together with rapsyn on the cell surface, low-affinity IgG1 antibodies were detected in 25 of 38 sera that had previously been negative in solution-phase assays; the antibodies could activate complement (22). Subsequent clinical work confirmed the diagnostic value of clustered AChR antibodies in conventionally seronegative MG (23). Antigen presentation can therefore change not only analytical sensitivity but also the biological meaning of an antibody signal.

Agrin is technically more difficult. The 2021 Chinese study used full-length agrin expressed in HEK293T cells, potentially broadening epitope representation compared with truncated constructs (12). However, the cells were fixed and permeabilized before serum incubation. Full-length antigen presentation should therefore not be equated with physiological extracellular access to agrin at the intact NMJ. The assay demonstrates reactivity to the expressed protein, not necessarily the geometry of antibody-target engagement *in vivo*.

The issue is sharper for intracellular antigens. Cortactin and rapsyn can be detected in permeabilized cells, recombinant-protein assays, or immunoblots, but such systems remove anatomical barriers faced by circulating IgG. Positivity in these assays establishes immunoreactivity; it does not, by itself, establish native target engagement at an intact synapse.

LRP4 provides the converse example. It is a surface-accessible antigen with substantial experimental support for pathogenicity, yet clinical detection remains assay dependent. In a 2026 multicentre study, LRP4-IgG positivity showed limited concordance between methanol-fixed and paraformaldehyde-fixed CBAs, and no samples were positive by the live CBA used in that study (8). This does not negate the experimental biology of LRP4 autoimmunity; it shows that assay performance and biological pathogenicity answer different questions.

### 2.3 Native antigen accessibility constrains the direct pathogenic hypothesis

AChR, MuSK, and LRP4 expose extracellular domains on the postsynaptic membrane, while neural agrin and ColQ occupy extracellular synaptic compartments (**Figure 2**). Circulating immunoglobulin can therefore reach these targets without requiring cellular entry or tissue disruption. Accessibility is not evidence of pathogenicity, but it is a basic biological requirement for a conventional direct antibody mechanism.

**Figure 2:**
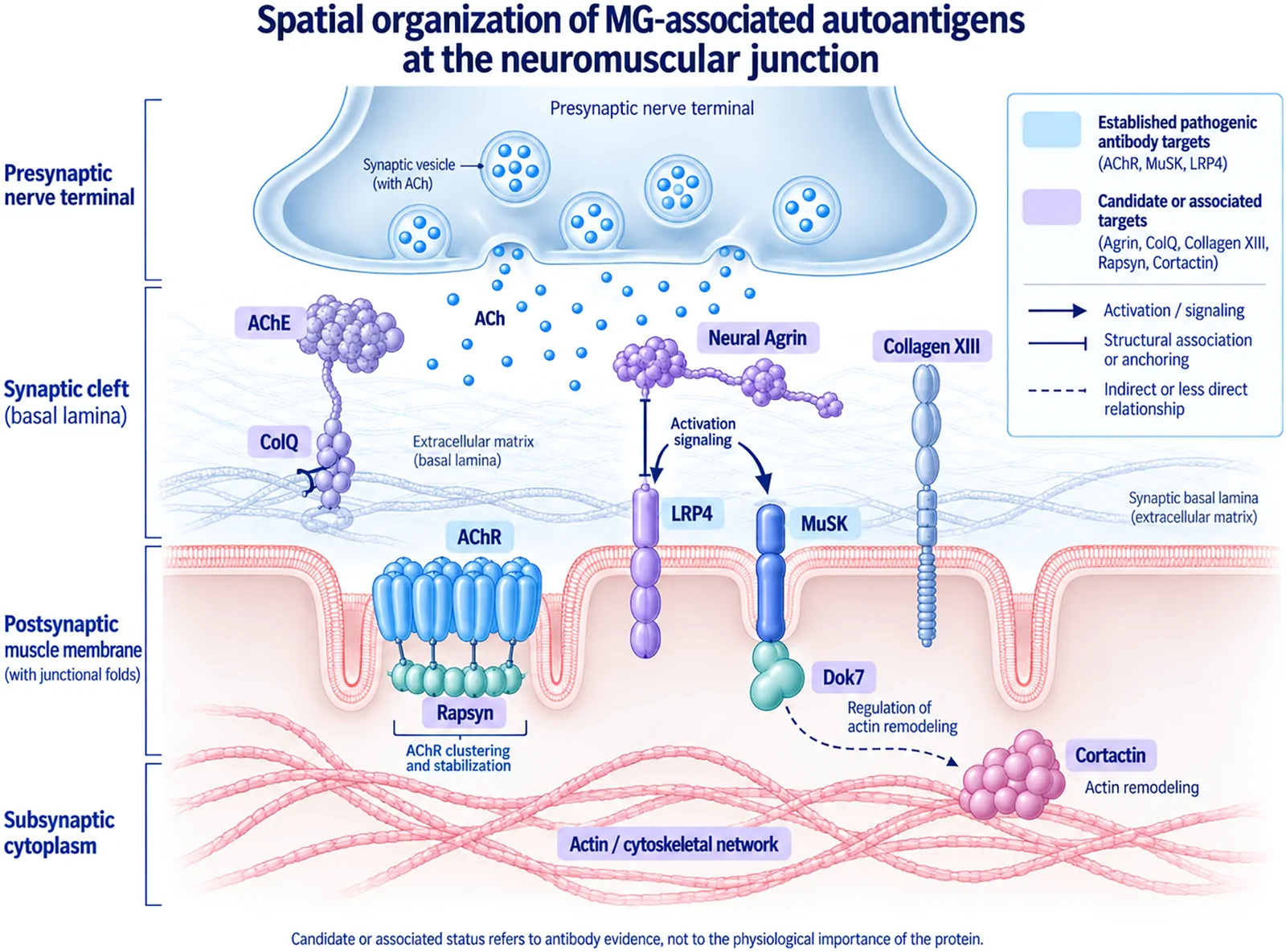
Spatial organization of MG-associated autoantigens at the neuromuscular junction. AChR, MuSK, and LRP4 have substantial evidence for pathogenic antibody effects, whereas agrin, ColQ, collagen XIII, rapsyn, and cortactin have differing unresolved antibody-level causal questions. The diagram distinguishes extracellular/surface-exposed proteins from intracellular/subsynaptic pools and depicts the agrin–LRP4–MuSK–Dok7 pathway and postsynaptic scaffolding. Extracellular localization makes antibody binding anatomically plausible but does not establish effective exposure of the relevant native epitopes, *in vivo* access, or pathogenic engagement. The schematic is conceptual and not to scale.

For intracellular targets, the mechanistic burden is different. Cortactin participates in actin remodeling associated with AChR cluster formation, while rapsyn is a postsynaptic scaffold essential for AChR clustering and stabilization. Their importance to NMJ biology is not in question. What remains uncertain is whether circulating antibodies can engage the relevant native protein pools in living muscle.

Intracellular localization does not make an antibody biologically irrelevant. It does, however, leave an additional causal step unresolved. Intracellular antigens may become exposed after tissue injury, an antibody may mark broader loss of tolerance, or less conventional mechanisms of antibody uptake may exist. These possibilities remain hypotheses until target engagement is demonstrated under disease-relevant conditions.

## 3 From target engagement to neuromuscular junction dysfunction

### 3.1 Mature causal benchmarks: AChR and MuSK

AChR antibodies provide a mature example of convergent causal evidence. Patient immunoglobulin can reproduce characteristic electrophysiological and postsynaptic abnormalities after passive transfer, and defined monoclonal antibodies can induce receptor loss and experimental weakness (2, 24). Molecular effects such as receptor cross-linking, internalization, direct functional interference, and complement-mediated postsynaptic injury can be linked to impaired neuromuscular transmission. Recent longitudinal profiling has added another layer: among 50 AChR-IgG-positive patients, complement activation, receptor internalization, binding-site blockade, isotype, and subclass features were heterogeneously distributed and changed over time (25). Thus, even for an established target, antigen specificity alone does not capture the full pathogenic phenotype of the antibody repertoire. The purpose of AChR in this Review is not to rehearse these established mechanisms, but to define the level of convergent evidence against which newer specificities are interpreted.

MuSK provides a complementary benchmark because pathogenicity need not depend on classical complement injury (32). Patient-derived MuSK IgG4 can inhibit the LRP4-MuSK interaction and agrin-stimulated MuSK phosphorylation (26, 27). Monoclonal studies further show that pathogenic behavior depends not only on antigen specificity but also on antibody architecture: monovalent Fab fragments derived from patient MuSK antibodies inhibit MuSK phosphorylation and AChR clustering, whereas corresponding bivalent antibodies can activate MuSK (3). Functional monovalency produced by Fab-arm exchange further amplifies pathogenicity *in vivo* (4). Antigen recognition alone therefore does not predict biological effect.

### 3.2 Molecular perturbation should lead to disease-relevant physiology

A candidate antibody becomes more persuasive when its effect matches the known biology of the target. For agrin, the relevant pathway is well defined: neural agrin binds LRP4, promotes MuSK activation, and initiates postsynaptic differentiation leading to AChR clustering. Anti-agrin sera from MG patients have been shown to inhibit agrin-induced MuSK phosphorylation and AChR clustering in muscle cells (10). Active immunization against neural agrin in mice produces weakness, abnormal NMJ morphology, and impaired endplate physiology (19).

Biochemical or structural readouts remain intermediate outcomes because MG is ultimately a disorder of neuromuscular transmission. Stronger evidence links molecular perturbation to endplate potentials, repetitive stimulation decrement, compound muscle action potentials, contractile force, calcium responses, or activity-dependent transmission failure. The LRP4/agrin passive-transfer study is informative in this respect because mixed patient Ig produced weakness, reduced CMAPs, impaired muscle force, abnormal endplate physiology, and structural fragmentation of the NMJ (20). These functional readouts move the evidence beyond target binding alone, although they do not by themselves resolve which antibody within a mixed IgG preparation is responsible.

The same logic prevents a common inferential error for candidate antigens: the physiological importance of a protein does not establish pathogenicity of an antibody directed against that protein. This distinction is especially relevant to NMJ proteins whose loss of function is already known to impair synaptic transmission.

## 4 Establishing antibody-specific causality

Demonstrating that patient immunoglobulin perturbs neuromuscular transmission does not necessarily identify the responsible specificity. Serum, total immunoglobulin, and purified IgG are not interchangeable preparations: their composition must be reported precisely. Whole immunoglobulin preparations may contain IgG together with other immunoglobulin classes, whereas IgG purification excludes them. A phenotype induced by a preparation containing several antigen reactivities establishes activity of that preparation, not automatically the sufficiency of any single specificity.

Antigen-affinity purification narrows the attribution problem but introduces practical constraints, including yield, retention of native affinity, and incomplete representation of a polyclonal response. Patient-derived monoclonal antibodies allow epitope, isotype, valency, and Fc-dependent effects to be tested in a defined manner. This does not make monoclonal antibodies more physiologically representative than total patient IgG; it makes them more causally interpretable. A recent review of monoclonal passive-transfer experimental autoimmune myasthenia gravis (EAMG) emphasizes this distinction (28).

These preparations answer different causal questions rather than forming a ladder of biological realism. The critical domains are (i) reproducible disease-associated serology, (ii) native target accessibility and engagement, (iii) antigen-linked molecular perturbation, (iv) impaired neuromuscular transmission, and (v) specificity-resolved causal attribution. Evidence of a biological effect on transfer must be distinguished from proof that an individual antibody specificity is sufficient. Antigen-specific affinity purification with selective immunodepletion and reconstitution can test contribution and restoration of function; monoclonal antibodies can test defined sufficiency. Both approaches can be combined with passive transfer and physiological experiments, and none is inherently more representative of the native polyclonal response.

Negative evidence also requires mechanistic interpretation. Failure of passive transfer may reflect species-specific epitope differences, inadequate target expression, mismatch between human IgG subclasses and murine Fc/complement biology, insufficient exposure, or absence of a required cofactor. A negative experiment is most informative when the model is demonstrably capable of reproducing the proposed mechanism. Repeated failure across complementary systems, particularly after confirmed native target engagement, should progressively weaken a direct pathogenic hypothesis.

## 5 Three instructive cases

### 5.1 LRP4: pathogenicity established, mechanism still being resolved

LRP4 has substantial experimental support as a pathogenic antibody target: receptor-directed immunization and transfer experiments support an effect on neuromuscular function (5, 6). We therefore distinguish evidence supporting pathogenic potential at the target level from the stronger claim that pathogenicity has been demonstrated for each isolated clinical LRP4-IgG result. The former is supported experimentally; the latter requires assay confirmation, clinical concordance, and, for specific downstream mechanisms, antibody-specific functional evidence. This distinction reconciles the target-level description with the evidence-domain assessment in **Table 1**.

**Table 1:** Current causal evidence and unresolved questions for MG-associated autoantibodies evidence-domain labels distinguish target-level experimental support from patient-level antibody attribution; “established target” does not mean every clinical positive result is independently pathogenic.

Autoantibody	Serological evidence	Native target accessibility	Antibody-specific functional evidence	Causal attribution	Principal unresolved question
AChR	Established	Postsynaptic surface; directly accessible	Established	Established	Relative contribution of individual effector mechanisms
MuSK	Established	Postsynaptic surface; directly accessible	Established	Established	Epitope-, subclass-, and valency-dependent effects
LRP4	Established; assay-dependent detection	Postsynaptic surface; directly accessible	Supported in experimental models; human specificity-dependent mechanisms incompletely resolved	Supported at target level; isolated clinical positives require confirmation	Clinical assay concordance; LRP4-specific complement attribution
Agrin	Repeatedly reported; assay-dependent prevalence	Synaptic extracellular compartment; accessible	Supported	Unresolved for isolated human anti-agrin antibody	Immunodepletion/reconstitution or defined monoclonal sufficiency
Cortactin	Inconsistent	Intracellular/subsynaptic; access unresolved	Not established	Not established	Native target access and direct NMJ dysfunction
ColQ	Limited	Synaptic basal lamina; accessible	Not established	Not established	Disease specificity and direct functional effect
Rapsyn	Historical/limited	Intracellular/subsynaptic; access unresolved	Not established	Not established	Native accessibility and modern independent validation
Collagen XIII	Limited	Postsynaptic membrane with extracellular domain	Not established	Not established	Replication and direct physiological effect

The remaining questions concern assay robustness and effector biology. Functional profiling in 2024 detected antibody-dependent cellular cytotoxicity (ADCC) and antibody-dependent cellular phagocytosis, with relatively weak complement deposition compared with AChR antibodies and no substantial rise in circulating activated complement proteins (7). In a 2026 biopsy series, C5b-9 deposition was observed at the NMJ of all seven LRP4-antibody-positive cases, with heterogeneous endplate involvement and IgG1 colocalization (9). However, this small observational series included three patients with intermittent low-titer AChR-antibody positivity. Complement deposition establishes local complement-associated pathology in the examined cases; it cannot, on its own, assign that deposition specifically to anti-LRP4 antibodies or exclude contributions from other antibodies. The relative contribution of LRP4-mediated signaling blockade, Fc-dependent activity, and local complement injury therefore remains uncertain.

LRP4 illustrates why experimental target-level pathogenicity and diagnostic interpretation of a patient-level result must remain distinct. In a 2026 multicentre comparison, positivity depended on cell-based assay format, with poor concordance across fixation conditions and no positives by the live assay examined (8). An isolated low-titer LRP4 result, particularly when obtained from one platform, should not be treated as definitive proof of MG or as proof of a particular effector pathway. Compatible clinical and electrophysiological findings, conventional AChR/MuSK serology, and orthogonal or reference-laboratory confirmation are important when the result would alter diagnosis or management. This diagnostic limitation does not nullify the experimental evidence of LRP4-directed pathogenic effects.

### 5.2 Agrin: a causal-attribution problem

Agrin is a biologically compelling candidate pathogenic specificity because it occupies a central extracellular position in postsynaptic organization. Neural agrin binds LRP4 and promotes MuSK activation, making direct antibody interference anatomically and mechanistically plausible. Its unresolved status reflects incomplete antibody-specific attribution rather than lack of biological relevance.

Serological evidence is heterogeneous. Anti-agrin antibodies were identified in 7 of 93 MG patients in one 2014 cohort, including two of four cases negative for AChR, MuSK, and LRP4 antibodies (termed triple-seronegative in that study), and were absent from 80 controls (10). Another 2014 study identified agrin antibodies only in combination with other MG antibodies (11). The large 2021 Chinese cohort detected agrin antibodies in 18 of 1,948 patients, including nine agrin-only positive cases (12). In contrast, an independent 2021 study of 135 MG patients detected none (13). Differences in patient selection and assay architecture complicate prevalence comparisons.

Functional evidence is stronger than prevalence estimates alone. Patient sera containing agrin antibodies can inhibit agrin-induced MuSK phosphorylation and AChR clustering (10). Active immunization against neural agrin can produce weakness and NMJ abnormalities in mice (19), although active immunization does not isolate the effect of human anti-agrin antibodies. Importantly, the passive-transfer experiment in reference 15 used purified patient immunoglobulin (Ig), containing IgG, IgA, and IgM, from a patient reactive to both LRP4 and agrin—not an IgG-only or anti-agrin-specific fraction. Transferred patient Ig caused weakness, altered electrophysiology, reduced muscle force, and NMJ fragmentation, and inhibited agrin-dependent signaling (20). These findings show pathogenic activity of that mixed preparation but cannot distinguish anti-agrin from anti-LRP4 contributions.

Independent sufficiency of human anti-agrin antibodies remains unresolved because the transferred immunoglobulin included more than one immunoglobulin class and antigen specificity (20). A particularly informative design would compare the original preparation with the same sample after selective anti-agrin immunodepletion and then reconstitute the depleted preparation with the recovered anti-agrin fraction, using matched controls and quantitative verification of depletion. Reciprocal anti-LRP4 depletion would help separate contributions and possible synergy. Defined patient-derived anti-agrin monoclonal antibodies offer a complementary sufficiency experiment; all preparations should be linked to signaling, NMJ physiology, and, where feasible, a transfer phenotype.

### 5.3 Cortactin: an accessibility and pathogenicity problem

Cortactin contributes to actin remodeling associated with AChR cluster formation. Experimental work has shown cortactin enrichment at AChR clusters and a role in agrin-induced postsynaptic organization (21). This establishes biological relevance of the protein, not pathogenicity of an antibody directed against it.

Serological studies have produced inconsistent results. The initial report found antibodies in 19.7% of seronegative MG, with lower frequencies in seropositive MG, other autoimmune diseases, and healthy controls (14). A subsequent study suggested enrichment among double-seronegative patients (15). The later independent cohort found only three positive MG patients and two positive controls, with no persistence in follow-up sera (13).

The more fundamental problem is topological. Cortactin is predominantly intracellular and subsynaptic. Detection after cell permeabilization, protein extraction, or immunoblotting demonstrates immunoreactivity but does not show that circulating IgG engages native cortactin at an intact NMJ. A conventional direct antibody mechanism would therefore require evidence of native target access, followed by target-specific perturbation and physiological transmission failure. That causal chain has not yet been demonstrated.

Anti-cortactin immunity may still be clinically or biologically informative. It could mark broader loss of tolerance, arise after exposure of intracellular antigen during tissue injury, or identify a patient subgroup without directly causing weakness. Current data do not distinguish these possibilities. A direct pathogenic role remains unproven, not excluded.

## 6 Other unresolved candidate targets

ColQ, rapsyn, and collagen XIII are most useful as boundary cases because they remain unresolved for different reasons.

ColQ is located in the synaptic basal lamina and is essential for anchoring the collagen-tailed form of acetylcholinesterase. Its extracellular location makes direct antibody binding anatomically plausible. In the largest screening study, ColQ antibodies were detected in 12 of 415 MG sera and one of 43 controls; five positive patients also had AChR antibodies and two had MuSK antibodies. The frequency did not differ significantly from the small control group, and direct functional effects were not demonstrated (16). ColQ therefore represents an accessible antigen with limited disease specificity and no established antibody-specific causality.

Rapsyn is a postsynaptic scaffold central to AChR clustering and stabilization, but the relevant protein pool is intracellular/subsynaptic. Rapsyn antibodies were reported historically (17), yet modern evidence linking isolated anti-rapsyn antibody to native target engagement and neuromuscular transmission failure is lacking. Rapsyn is a particularly clear example of the distinction between pathogenic relevance of a protein and pathogenicity of an antibody directed against that protein.

Collagen XIII is a transmembrane collagen enriched at the NMJ, with an extracellular domain extending into the synaptic compartment. Autoantibodies to collagen XIII have been reported in MG (18), but independent replication and direct physiological studies remain insufficient. It should therefore be treated as a reported MG-associated target rather than an established pathogenic antibody.

These examples remain unresolved at different points in the causal chain. ColQ is accessible but weakly validated as disease specific; rapsyn is functionally central but topologically difficult for circulating IgG; and collagen XIII is anatomically plausible but mechanistically underdeveloped. Treating all three as equivalent “novel pathogenic antibodies” would blur rather than clarify those differences. **Table 1** summarizes the current evidence and the principal unresolved question for each antibody specificity.

## 7 Clinical utility without direct pathogenicity

Not all antibodies detected in MG need to cause the core transmission defect. Anti-titin antibodies are striational markers commonly associated with thymoma-associated MG and can also occur in late-onset non-thymomatous MG; their presence is not specific for thymoma. Ryanodine receptor (RyR) antibodies have been reported particularly in thymoma-associated MG and have associations with more severe or predominantly bulbar disease in some cohorts. Kv1.4 antibodies have been associated with particular MG subgroups and, notably, with myocarditis or other cardiac involvement in some series, although frequency and predictive value vary by population (29, 30). None should be interpreted as a stand-alone diagnostic test for thymoma, severity, or cardiac disease. Their chief use is adjunctive clinical stratification, not established direct interruption of neuromuscular transmission.

### 7.1 Interpreting expanded antibody panels in clinical practice

Expanded MG antibody panels require interpretation at two separate levels: biological evidence for the target and the validity of an individual laboratory result. For AChR and MuSK, validated serology can usually be interpreted alongside established mechanisms. LRP4-directed antibodies have experimental pathogenic support, but assay discordance means an isolated LRP4-positive result—especially a weak positive from one fixed-cell platform—requires particular caution (8). A clinically compatible pattern of fatigable weakness, appropriate electrophysiology, exclusion of alternative diagnoses, and confirmation with an independent assay where available should govern its weight. For agrin, cortactin, ColQ, rapsyn, and collagen XIII, associations are less mature and results should not independently establish MG or trigger antigen-specific treatment. Conversely, a biomarker may still be useful for phenotype stratification without having proved direct NMJ pathogenicity. Clinical utility, analytical performance, and mechanistic causality must be reported as different properties.

## 8 Experimental design should follow the missing link

### 8.1 If the problem is serological reliability

Experimental design is most informative when it is matched to the unresolved causal step. **Table 2** summarizes five practical questions that can be used to identify that step. When serological reliability is the main uncertainty, independent replication with appropriately matched neurological and autoimmune controls is the priority, ideally using an orthogonal assay. For low-frequency antibodies, antigen length, conformation, fixation, permeabilization, membrane presentation, and positivity thresholds should be reported explicitly. Reproducibility across cohorts is most informative when the assay itself is biologically interpretable.

**Table:** Five questions for evaluating a newly reported MG-associated autoantibody.

Question	Interpretive purpose
Is the antibody signal independently reproducible in MG and substantially weaker or absent in appropriate controls?	Tests whether the serological association is reproducible and disease related rather than assay- or cohort-specific.
Can circulating antibody engage the native antigen under physiologically relevant conditions?	Distinguishes antibody detection from physiologically plausible engagement of the native target.
Does antigen-specific antibody produce the expected molecular or structural perturbation?	Tests whether antigen-specific antibody produces the molecular or structural effect predicted from target biology.
Does that perturbation impair neuromuscular transmission?	Links target perturbation to the defining physiology of MG rather than to an intermediate biochemical readout alone.
Can the disease-relevant phenotype be attributed to the defined antibody specificity rather than to an unresolved polyclonal repertoire?	Separates pathogenicity of a mixed antibody repertoire from independent sufficiency of a defined antibody specificity.

### 8.2 If the problem is native antigen accessibility

When native accessibility is uncertain, live-cell or native-tissue binding is more informative than permeabilized-cell assays for a conventional direct antibody mechanism. For intracellular candidates such as cortactin or rapsyn, positive immunoblots should be interpreted as evidence of autoreactivity rather than *in vivo* target engagement. If a non-classical access mechanism is proposed, that mechanism should be tested directly.

### 8.3 If the problem is molecular or physiological function

Affinity-purified or recombinant patient-derived antibodies can test whether target engagement produces the expected molecular perturbation. The experiment should then connect that perturbation to neuromuscular transmission. Relevant readouts include MuSK phosphorylation, AChR clustering, endplate potentials, CMAP decrement, contractile force, calcium handling, and activity-dependent failure. Human motor neuron-muscle cocultures and microphysiological NMJ systems can provide an intermediate level of validation when they preserve human antigen biology and functional synaptic readouts. A human microfluidic NMJ model first published online in 2025, for example, reproduced anti-AChR antibody-mediated complement activation and transmission dysfunction, illustrating how human systems can connect molecular mechanism to physiological output (31). Their value depends on the causal question they are used to answer, not on technical complexity alone.

### 8.4 If the problem is antibody-specific attribution

When antibody-specific attribution is the limiting step, passive transfer of a mixed immunoglobulin preparation does not identify the active specificity. Antigen-selective immunodepletion followed by restoration of the depleted antigen-specific fraction (reconstitution) can test whether the candidate antibody is necessary for the observed effect and whether that effect can be rescued. Reciprocal depletion is particularly informative when both agrin and LRP4 reactivities coexist. Affinity-purified fractions and patient-derived monoclonal antibodies can then test sufficiency and mechanism, using native-antigen binding, MuSK phosphorylation/AChR clustering, and physiological transmission or transfer readouts. These approaches are complementary, not mutually exclusive or arranged in a fixed hierarchy (20, 28).

### 8.5 If pathogenicity is established but mechanism remains uncertain

Once pathogenicity is reasonably established, the experimental question changes. Repeating generic passive-transfer studies becomes less informative than resolving epitope, subclass, Fc function, tissue pathology, and pathway-specific rescue. LRP4 is the clearest example. Complement inhibition would test a complement-dependent component, whereas interventions that restore LRP4-MuSK signaling would test functional blockade. Rescue is not a mandatory criterion for pathogenicity, but mechanism-matched rescue can provide strong additional support for a proposed pathway.

## 9 Discussion and future directions

The central argument of this Review is that MG-associated autoantibodies should not be interpreted along a single axis from “negative” to “pathogenic.” Distinct uncertainties can persist at different stages of the evidence chain. An assay may detect a reproducible antibody while native target access remains uncertain; patient IgG may impair neuromuscular transmission while the responsible specificity remains unresolved; or direct pathogenicity may be established while the dominant downstream effector mechanism remains heterogeneous. These are experimentally different problems and should not be collapsed into a single evidence tier.

Unlike descriptive accounts of MG antibody subtypes, this review separates five independently assessable evidence domains and identifies the limiting inference for each specificity (**Table 1**). For example, the main unresolved LRP4 questions concern reproducibility of clinical detection and attribution of downstream complement injury, whereas agrin requires experiments isolating the human anti-agrin fraction, and cortactin first requires demonstration of access to native intracellular/subsynaptic antigen. This comparison is intended as an experimental decision aid rather than a numerical ranking of antibodies.

Priorities should be selected by the missing causal step rather than by a rigid technology ladder. For inconsistent serology, use shared specimen panels, independent cohorts, explicit thresholds, and orthogonal tests. For uncertain accessibility, test binding in live cells or intact native tissue. For mechanistic attribution, use selective depletion/reconstitution, purified fractions, and defined monoclonal antibodies with functional readouts; for a suspected complement pathway, couple tissue observations to antibody-specific complement assays and pathway-matched perturbation or rescue. Human neuromuscular-junction systems complement, rather than replace, physiologically relevant transfer models.

This framework remains interpretive rather than a prospectively validated scoring instrument. The five evidence domains overlap and can be tested in different orders; no individual experiment guarantees clinical relevance or species transferability. A negative transfer result is difficult to interpret if epitopes, Fc receptors, complement biology, or antigen expression are mismatched. Conversely, fixed/permeabilized assays can overstate antigen access, and mixed immunoglobulin preparations can obscure the causal specificity. These limitations require transparent reporting, not automatic downgrading or upgrading based on one test.

## 10 Conclusion

The expanding autoantibody repertoire of MG has outpaced mechanistic validation of many newly reported reactivities. AChR and MuSK remain mature examples in which serological association, molecular effect, physiological dysfunction, and experimental causality converge. LRP4 is also supported as a pathogenic target, whereas agrin, cortactin, ColQ, rapsyn, and collagen XIII remain unresolved at different points in the causal chain.

The most useful question is therefore not whether an antibody is simply “novel” or “established,” but which causal step has been demonstrated and which remains uncertain. Matching experimental design to that missing step should improve interpretation of expanded antibody panels and help determine which serological signals ultimately become part of the disease mechanism.
